# Supplementary material for: A Multistage Formulation Based on Full-Length CSP and AMA-1 Ectodomain of Plasmodium vivax Induces High Antibody Titers and T-cells and Partially Protects Mice Challenged with a Transgenic Plasmodium berghei Parasite
Source: Microorganisms. 2020 Jun 17;8(6):916. doi: 10.3390/microorganisms8060916 (PMC7356588; doi:10.3390/microorganisms8060916)
Supplement: Supplementary file 1 [file microorganisms-08-00916-s001.pdf]

# Supplementary figure 1

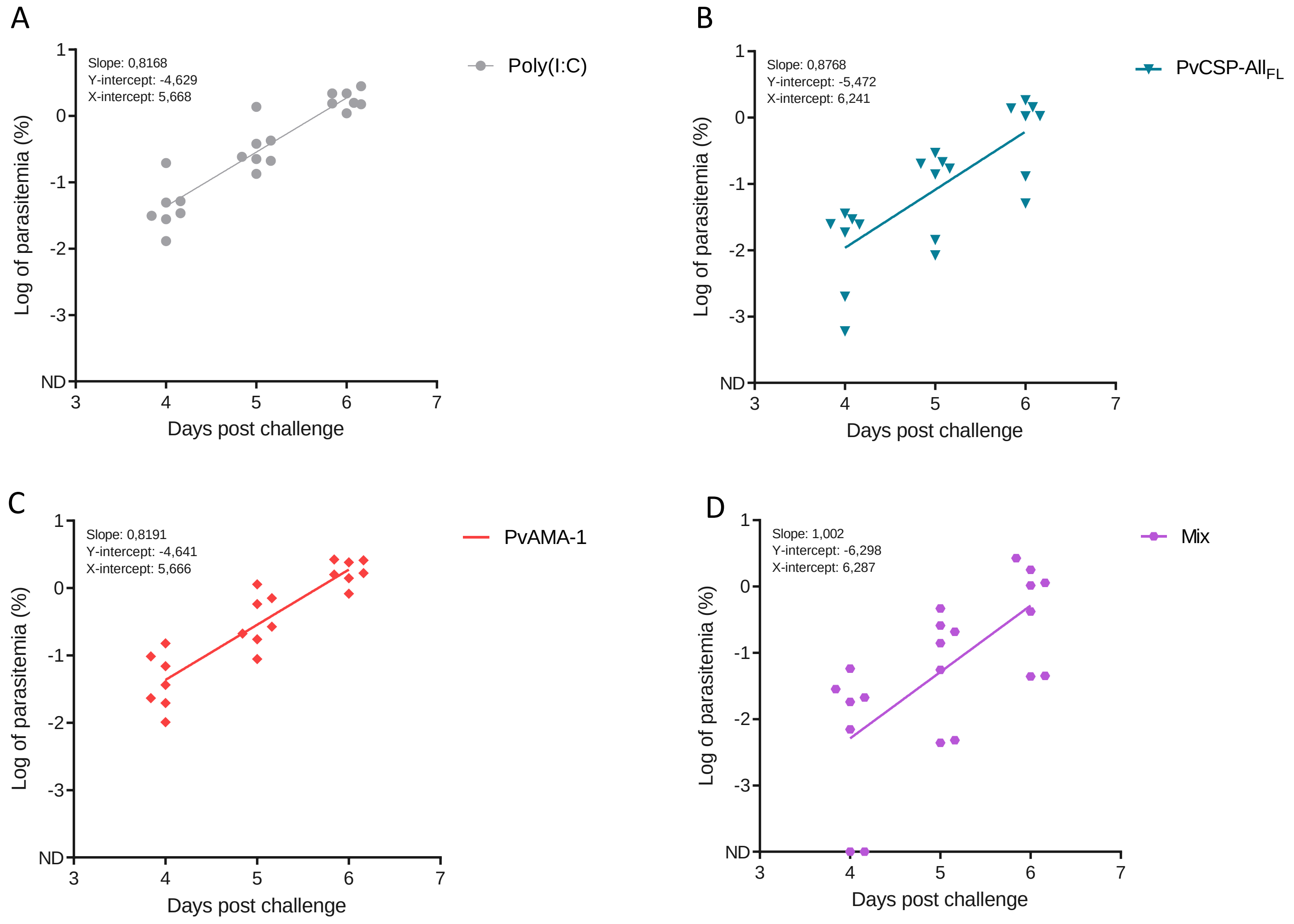


**Supplementary Figure 1. Evaluation of parasitemia after challenge in immunized mice**. Groups of seven 6- to 8-week-old female C57BL/6 mice were immunized (s.c.) with Poly(I:C) (50 µg/dose) **(A)**, plus 10 µg of PvCSP-All$_{FL}$ **(B)**, 10 µg of PvAMA-1 **(C)** or the Mix of 10 µg of each recombinant protein **(D)**. On day 58 after priming, the mice were challenged with 5,000 Pb/PvCSP-VK210 transgenic sporozoites as described in materials and methods. Parasitemia was analyzed by flow cytometry. The percentage of infected red blood cells (iRBCs) on days four, five, and six post-infection (p.i.) was expressed as log values for normalization before statistical analysis. The log of parasitemia post-challenge was measured on each immunized group and slopes and intercepts used to calculate p-values are indicated in the graphs.
